# Influence of *in situ* progressive N-terminal is still controversial truncation of glycogen branching enzyme in *Escherichia coli* DH5α on glycogen structure, accumulation, and bacterial viability

**DOI:** 10.1186/s12866-015-0421-9

**Published:** 2015-05-07

**Authors:** Liang Wang, Ahmed Regina, Vito M Butardo, Behjat Kosar-Hashemi, Oscar Larroque, Charlene M Kahler, Michael J Wise

**Affiliations:** School of Pathology and Laboratory Medicine, University of Western Australia, Perth, Australia; CSIRO Agriculture Flagship, Canberra, Australia; International Rice Research Institute, Los Baños, Philippines; School of Chemistry and Biochemistry, University of Western Australia, Perth, Australia

**Keywords:** Glycogen structure, Microbial abiotic stress tolerance

## Abstract

**Background:**

Glycogen average chain length (ACL) has been linked with bacterial durability, but this was on the basis of observations across different species. We therefore wished to investigate the relationship between bacterial durability and glycogen ACL by varying glycogen average chain length in a single species. It has been shown that progressive shortening of the N-terminus of glycogen branching enzyme (GBE) leads to a lengthening of oligosaccharide inter-α-1,6-glycosidic chain lengths, so we sought to harness this to create a set of *Escherichia coli* DH5α strains with a range of glycogen average chain lengths, and assess these strains for durability related attributes, such as starvation, cold and desiccation stress resistance, and biofilm formation.

**Results:**

A series of *Escherichia coli* DH5α mutants were created with *glgB* genes that were *in situ* progressively N-terminus truncated. N-terminal truncation shifted the distribution of glycogen chain lengths from 5-11 DP toward 13-50 DP, but the relationship between *glgB* length and glycogen ACL was not linear. Surprisingly, removal of the first 270 nucleotides of *glgB* (glgBΔ270) resulted in comparatively high glycogen accumulation, with the glycogen having short ACL. Complete knockout of *glgB* led to the formation of amylose-like glycogen containing long, linear α1,4-glucan chains with significantly reduced branching frequency. Physiologically, the set of mutant strains had reduced bacterial starvation resistance, while minimally increasing bacterial desiccation resistance. Finally, although there were no obvious changes in cold stress resistance or biofilm forming ability, one strain (glgBΔ180) had significantly increased biofilm formation in favourable media.

**Conclusions:**

Despite *glgB* being the first gene of an operon, it is clear that *in situ* mutation is a viable means to create more biologically relevant mutant strains. Secondly, there was the suggestion in the data that impairments of starvation, cold and desiccation resistance were worse for the strain lacking *glgB*, though the first of these was not statistically significant. The results provide prima facie evidence linking abiotic stress tolerance with shorter glycogen ACL. However, further work needs to be done, perhaps in a less labile species. Further work is also required to tease out the complex relationship between glycogen abundance and glycogen structure.

**Electronic supplementary material:**

The online version of this article (doi:10.1186/s12866-015-0421-9) contains supplementary material, which is available to authorized users.

## Background

Glycogen is a major intracellular carbon and energy reserve in microorganisms, which is normally accumulated when a carbon source is abundant while other nutrients are deficient [1]. It is a hyperbranched homopolysaccharide consisting of only glucosyl residues, which were linked together by α-1,4-glycosidic bonds in linear chains and α-1,6-glycosidic bonds at branching points [1]. Currently, there are more than 50 bacterial species reported to store glycogen [2] and 245 bacterial species harboring the essential genes for glycogen metabolism [3]. The order of these genes in the common *glg* operon is not always consistent [4] and the precise role for glycogen in bacteria is still not clearly understood [5]. Previous studies have linked glycogen with bacterial starvation survival [6], environmental persistence and transmission [5], and symbiotic performance [7], though its role in bacterial colonization and virulence is still controversial [8-10]. In addition, Pan et al. [11] reported that trehalose synthase (TreS) converts glycogen to trehalose. Chandra et al. [12] also identified a widespread non-classical GlgE pathway, converting trehalose to α-glucan (glycogen). A connection between glycogen and trehalose may extend the function of glycogen to bacterial cold and desiccation resistance due to the protective role of trehalose under these stresses [13,14].

Although the role of glycogen in bacteria is still under investigation, according to a recent review, glycogen structure, specifically average chain length (ACL) – the average number of 1,4-glycosidic-bonded glucosyl units between 1,6-glycosidic- bonded glucosyl units – may play an important role in bacterial durability [3]. However, only a few biological studies and theoretical analyses are currently available to support this proposal [3]. In order to test this hypothesis experimentally, a set of bacterial strains from the same species accumulating glycogen with different ACLs was developed and their performance under a variety of conditions was compared. Five enzymes are considered to be core members of the glycogen metabolic pathway: glycogen synthase (GlgA, EC 2.4.1.21), ADP-glucose pyrophosphorylase (GlgC, EC = 2.7.7.27), glycogen branching enzyme (GBE) (GlgB, EC 2.4.1.18), glycogen phosphorylase (GlgP, EC 2.4.1.1), and glycogen debranching enzyme (GlgX, EC 3.2.1.-) [3,15]. A number of these genes influence bacterial inter-α-1,6-glycosidic chain-length distribution patterns: GlgB, GlgP and GlgX [16,17], and could therefore be starting points toward our aim of varying chain length distributions in a single species. It is known that *E. coli* GlgP can only act on linear chains longer than 4 glucosyl residues from the non-reducing end [16], while GlgX cleaves short oligosaccharides (up to 4 glucosyl residues) from α-1,6-branching points [17]. Accordingly, *glgP*-deficient cells accumulate glycogen with longer chain lengths while *glgX*-deficient cells have more glycogen with short-branched chains [16,17]. However, both *glgP* and *glgX* are involved in the glycogen degradation pathway and mutations would make bacterial cells unable to properly utilize glycogen. For example, *glgX*-deficient *Vibrio cholera* is observed to die faster under nutrient-limited conditions although more glycogen is accumulated than in the wild type strain [5]. In addition, over-accumulation of glycogen has protective roles against stresses such as low pH and osmotic stresses [5]. Of the other proteins involved in glycogen synthesis/metabolism, inactivation of GlgA leads to the loss of glycogen production [9,18], although a recent study indicated that there is an accessory pathway in *E. coli* that can utilize maltodextrin to synthesize glycogen in the absence of GlgA [19]. In addition, GlgC has a rate-controlling role by providing ADP-glucose for glycogen synthesis [15]. Thus, both GlgA and GlgC are not suitable for manipulating glycogen structure, leaving GlgB as the prime candidate for modification.

GBE belongs to GH13 family [20] and is involved in two processes: hydrolyzing α-1,4-glycosidic linkages and transferring oligosaccharide chains of mainly 5–16 glucosyl residues to a neighboring α-1,6-position [21]. Modification of the N-terminus of bacterial GBE can provide a practical approach to altering bacterial glycogen ACL quantitatively; an earlier study showed that proteolysis of the first 112 amino acids (AA) of *Escherichia coli* GBE changes glycogen chain length distribution patterns [22]. Later, Devillers et al. [23] reported that the length of the N-terminus of GBE is positively correlated with the length of the transferred chains. A recent experimental study also revealed that GBE N-terminus is responsible for substrate specificity and glycogen branching pattern by swapping N-terminal domains between *Deinococcus geothermalis* and *Deinococcus radiodurans* [20].

To date, all studies [20,23,24] involving the GBE N-terminus have been performed using purified protein and amylose as an artificial substrate, which may not reflect the real situation inside bacteria. To test the function of the N-terminus of GBE, a set of *E. coli* DH5α mutants with *in situ* progressive truncation of N-terminus of GBE were constructed. The influence of changing the length of the N-terminus of GBE on glycogen structure and accumulation was assessed. The effect of any changes in glycogen expression on bacterial growth under abiotic stresses, such as starvation, cold and desiccation, were assessed. Biofilm formation was also measured, considering its relationship with polysaccharide formation and bacterial persistence [8,25,26]. To the best of our knowledge, this is the first study of *in situ* N-terminal truncation of GlgB and its influences on glycogen structure, accumulation, and bacterial durability.

## Methods

### Bacterial strains, plasmids, and growth conditions

#### *E. coli* strains used in this study were as follows

DH5α, DH5α glgBΔ90, DH5α glgBΔ180, DH5α glgBΔ270, DH5α glgBΔ369, DH5α ΔglgB, BL21(DE3), JM109, DB3.1, and Top10 (Additional file 1: Table S1). All bacteria were cultured on agar containing 1×M9 salts (Sigma) supplemented with 1.5% agarose, 0.4% glucose, 0.2% thiamine, 2 mM MgSO4, and 0.1 mM CaCl2 for iodine vapor staining. Luria-Bertani agar plates contained antibiotics at the following concentrations: 100 μg/ml ampicillin, 50 μg/ml of kanamycin, or 25 μg/ml chloramphenicol. Unless otherwise stated, bacteria were cultured at 37°C incubator and shaken at 200 rpm in a rotary shaking incubator.

### Construction of in situ glgB mutated strains

**Figure 1:**
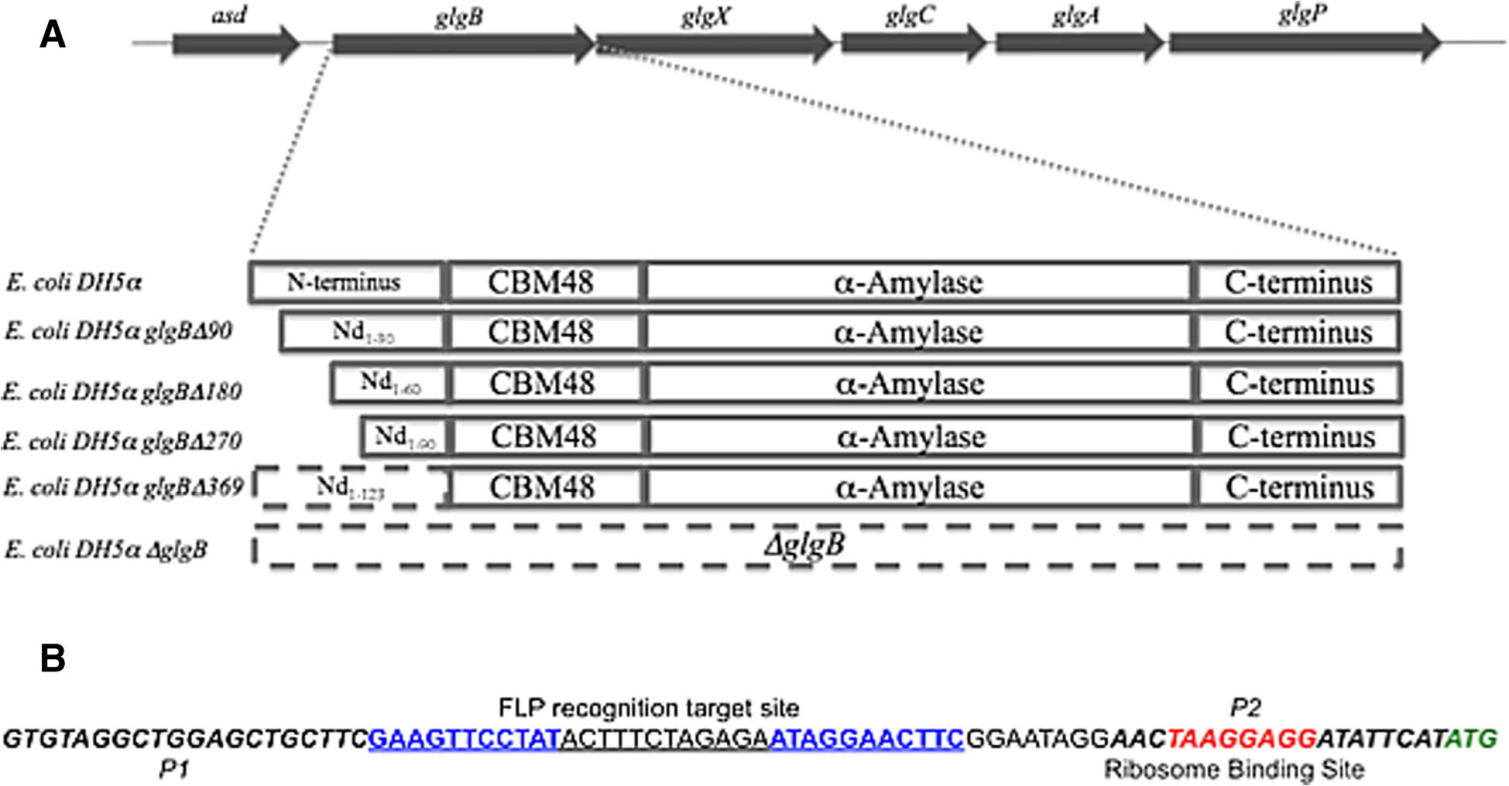
*GlgB* and *glgB*-mutated strains in their *E. coli* DH5α genomic context, and the scar sequence left following in situ mutation. **A**. The five essential glycogen metabolism genes are organized into a single transcriptional unit as *glgBXCAP* in *E. coli* DH5α (Montero et al. [44]). For GlgB in *E. coli* DH5α, four domains have been identified, which are N-terminus, CBM48, α-amylase, and C-terminus. **B**. P1 and P2 (italic letters) were primers for amplifying linear PCR products from plasmid pKD4. Underlined sequence was FRT site with a palindromic sequence (blue sequences), separated by 12 asymmetric nucleotides. Red italic sequence was a ribosome-binding site and the green italic letters represented start codon for downstream gene expression.

Five mutants of *E. coli* DH5α expressing progressively deleted GBE in the chromosomal position of *glgB* were constructed (Figure 1A). *E. coli* DH5α glgBΔ90 (4-90 nucleotides deleted), *E. coli* DH5α glgBΔ180 (4-180 nucleotides deleted), *E. coli* DH5α glgBΔ270 (4-270 nucleotides deleted), *E. coli* DH5α glgBΔ369 (4-369 nucleotides deleted), *E. coli* DH5α ΔglgB (4-2186 nucleotides deleted). λ-Red recombination system (Plasmids pKD4, pKD46, and pCP20) by Datsenko and Wanner [27] was generously provided by Dr. Harry Sakellaris. Plasmid pKD46 was first transformed into *E. coli* DH5α. Then, five pairs of primers with 36 nucleotides in 5′ and 3′ regions that correspond to homologous regions in *glgB* were used to amplify linear PCR products from plasmid pKD4. The list of primers can be found in Additional file 1: Table S2A, and the combinations of primers used for the particular deletion mutants can be found as Additional file 1: Table S2B. All the five linear PCR products had the length of 1.5 kb and contain a kanamycin resistance gene flanked by FRT sites. These linear PCR products were electroporated into competent *E. coli* DH5α cells carrying pKD46. Recombination catalyzed between the FRT sites and the *glgB* locus by the lambda red recombinase resulted in the replacement of the wild type *glgB* chromosomal locus with the deleted variants (Figure 1A).

Successful homologous recombination into the *glgB* locus with linear PCR products was verified by the existence of the kanamycin resistance gene in the chromosome. Primers K_t_ and K_2_ (Additional file 1: Table S2A) were used for verification [27]. A helper plasmid pCP20 encoding FRT recombinase was introduced into recombined strains, to facilitate the removal of the kanamycin resistance cassette by crossover of FRT sites. To confirm the loss of the kanamycin resistance cassette, flanking primers, FP*glgB* and RP*glgB* (Additional file 1: Table S2A), which covered the full *glgB* region, was used to verify the variation in the length of the locus. Finally, in order to make sure that no point mutations existed in the constructed mutants, PCR-based gene sequencing was done for the *glgB*-truncated region. Finally, plasmid pCP20 was removed from each of the recombinant strains by culturing at 37°C. Antibiotics sensitivity test was then performed on LB agar plate (100 μg/ml ampicillin) to confirm the loss of the plasmid.

### Quantitative reverse transcriptase real time PCR (qRT-PCR)

Expression levels of *glgB*, *glgX*, *glgC*, *glgA*, *glgP* were examined by qRT-PCR. Total RNA from stationary phase cells (20 hour) was extracted using the RiboPure™-Bacteria Kit (Invitrogen). Extracted RNA was then digested by DNaseI to reduce the risk of genomic DNA contamination. iScript^TM^ cDNA synthesis kit (Bio-Rad) was used to synthesize cDNA, following manufacturer’s instructions. Quantitative real-time PCR was set up (total of 20 μL reaction) by mixing RNA template, 2 × SensiFAST^TM^ SYBR&Fluorescein one-step mix, 10 μM forward primer, and 10 μM reverse primer. The PCR was performed in a Rotor-Gene 3000 PCR machine (Corbett Research) for 40 cycles with 2-step cycling, which consisted of denaturation at 95°C for 5 s and annealing at 60°C for 20s, with a final extension at 72°C for 1 minute. Melt curve analysis confirmed the presence of a single product from each PCR reaction (data not shown). The primer pairs used to assess the transcript levels were as follows: *glgB* was amplified using glgBF and glgBR; *glgX* was amplified using glgXF and glgXR; *glgC* was amplified using glgCF and glgCR; *glgA* was amplified using glgAF and glgAR and *glgP* was amplified using glgPF and glgPR (Additional file 1: Table S2A). The control gene, *cysG*, was amplified using cysGF and cysGR (Additional file 1: Table S2A). The relative changes in gene transcription were calculated using the comparative CT method, normalized to the level of *cysG* transcript. Each set of qRT-PCRs was examined in duplicate and was repeated with at least two independent RNA preparations.

### Iodine vapor staining to detect glycogen in bacterial cells

*E. coli* DH5α strains were cultured in 1 × M9 minimal agar plates for 48 hours before being exposed to iodine vapor for staining to detect glycogen [28,29]. A colony accumulating branched polysaccharide is stained brown while that with linear polysaccharide is stained blue due α-glucan helix complexation with iodine. Each agar plate was exposed to solid iodine flakes in a sealed glass beaker. The bottom of the beaker was heated inside the fume hood for about 1 min on a hot plate to generate iodine vapor. The plates were immediately removed when the colony color changed while the agar was still transparent (generally 30 seconds) to avoid over-staining.

### Glycogen extraction

Glycogen was extracted from *E. coli* DH5α strains by using a modified procedure described by Preiss et al. [30], which works equally well for both the branched molecule, glycogen, and its linear counterpart. *E. coli* DH5α was cultured in 1L 1 × M9 minimal medium at 37°C with gyratory shaking for 20 h. Cells were harvested by centrifugation at 5,000 × g, 4°C for 10 min (Sorvall, SLA3000). Pellets were resuspended in 20 ml of ice-cold 0.05 M TEA buffer and sonicated on ice for 3 min. Homogenized cell pellets were then centrifuged at 104,000 × g, 4°C for 90 min (Beckman, SW41Ti). Supernatant was discarded and pellets were resuspended in 20 ml MilliQ H_2_O. The resulting suspension was boiled in a water bath for 5 min to denature all proteins. The suspension was then centrifuged at 18,000 × g (Sorvall) for 10 min. The supernatant (S1) was kept. The precipitate was treated the same way and the new supernatant (S2) was pooled with S1. A 0.1 volume of 50% trichloroacetic acid (TCA) was added to the pooled supernatant (S1 + S2) in order to precipitate other macromolecules (DNA, RNA, and protein, etc.). The solution was placed on ice for 10 min, after which it was centrifuged at 18,000 × g for 10 min. The supernatant was collected and mixed with 1.5 volume of absolute ethanol. Glycogen was precipitated on ice for 20 min, after which it was centrifuged at 18,000 × g for 10 min. To purify the extracted glycogen, the pellet was dissolved in 5 ml MilliQ H_2_O and 5 ml ice-cold absolute ethanol was added. After incubating the solution at 4°C overnight, it was centrifuged at 18,000 × g for 10 min. The supernatant was discarded and pellet was kept. This wash/precipitation procedure was repeated two more times. Finally, the pellet was dissolved in acetone and left to completely air-dry at 37°C.

### Ultraviolet–visible spectroscopy (λ-max scanning)

Following the method of Nakamura et al. [31], 1 mg of glycogen extracted from each of the *E. coli* DH5α strains was weighed to make a 10 μg/μl glycogen solution. A serial dilution of 150 μL glycogen-iodine solution was prepared and transferred to a 96-well microplate. Blank controls were always kept, along with the test groups, by replacing glycogen solution with dH_2_O. A microplate reader was used to scan the absorbance of the samples from 350 nm to 700 nm.

### Glycogen content assay and comparison with protein content

Glycogen content was assayed for each of the six *E. coli* DH5α strains along their growth curves in 1×M9 minimal medium (T/G=1:2) with three independent replicates. Procedures were followed as described by Dauvillee et al [17]. Protein content was assayed by reference to a standard curve based on bovine serum albumin (0.25 mg/ml BSA and Coomassie Plus (Bradford)).

### Fluorophore-assisted carbohydrate electrophoresis (FACE) and ACL determination

FACE was used to analyze glycogen chain length distribution pattern. Glycogen was debranched as previously described [32]. The samples were dried in vacuo and reducing ends were labeled with the charged fluorophore 8-amino-1,3,6-pyrenetrisulfonic acid (APTS). Capillary electrophoresis was then used to resolve and detect the labeled oligosaccharides by increasing chain length. These procedures were performed according to Morell et al. [33]. The ACL of the glycogen was computed using the formula:

$$ \mathrm{A}\mathrm{C}\mathrm{L}=\frac{{\displaystyle \sum \mathrm{Length}\ \mathrm{of}\ \mathrm{Oligosaccharide}\ \mathrm{C}\mathrm{hain}\times \mathrm{Molar}\ \mathrm{Percentage}}}{100}. $$

### Reducing end assay

A maltotriose stock solution (1 mg/ml) was used to construct a maltotriose standard concentration curve (0 to 200 nmol), which was used as a reference for measuring reducing ends of 5 mg glycogen samples. Each sample was debranched as described above. Branching frequency estimation of debranched starch was determined based on a reducing end assay [34] as modified by [35].

### Starvation assay

*E. coli* DH5α strains were cultured in 1 × M9 minimal medium at 37°C for 20 hours with gyratory shaking. Cells were harvested by centrifugation at 5,000 × g for 10 minutes and washed in PBS buffer 3 times. In order to avoid the influence of cell lysis on bacterial starvation survival, cells were diluted 100 times with PBS buffer and left on the bench-top. After 0, 3, 6, 9, 13, and 15 days, colony-forming units (CFUs) for each strain were counted using the Miles and Misra method [36,37]. Two samples were measured for each strain at each time point.

### Desiccation assay

The desiccation assay was conducted on each of the *E. coli* DH5α strains by following the procedures as described in Walsh and Camilli [38], with modifications. In order to dry cells quickly in the laminar flow hood, the cells were concentrated 10 times in PBS buffer. A 15 μl culture aliquot was spread evenly on the lid of a sterile Petri dish and air-dried inside the hood. Samples were obtained at 0, 2, 3, 6, 9 hours by resuspending the dried cells in 1.5 ml PBS buffer. Viable cells were counted as stated above in starvation assay. Four samples were measured for each strain at each time point.

### Cold stress assay

Bacterial cultures were prepared the same way as for the desiccation experiments, except that the cell pellets were resuspended in 10 ml PBS buffer instead of being concentrating 10 times. The suspension was serially and aseptically diluted in a hood from 10^-1^ to 10^-8^ times by mixing 100 μl diluted culture with 900 μl PBS buffer. Since the original culture and 10^-1^ diluted culture had too high cell density, we started from 10^-2^ dilution until 10^-7^ dilution. Once the plates were dried, plates were wrapped into a sealed plastic bag and stored in a cold room (4°C). For cold viability measurement, plates were taken out of the cold room at day 0, 3, 6, 10, and 13 and incubated at 37°C overnight. The colony forming units were counted and calculated the next day. Each count was repeated 3 times.

### Biofilm formation assay

The procedure for biofilm formation ability was sourced from Merritt et al. [39], Narisawa et al. [40], and Burton et al. [41]. Greiner CELLSTAR® 96 well plates (polystyrene, flat bottom with lid, sterile) were used. The *E. coli* DH5α strains were cultured in LB and 1 × M9 minimal medium (T/G = 1:2). The samples were stained with crystal violet. Results were obtained from three independent replicates.

## Results

### Construction of in situ N-terminal truncated GlgB in E. coli DH5α

In order to construct bacterial strains with the same genetic background but accumulating differential ACL glycogen, a suite of *E. coli* DH5α strains with N-terminal progressively truncated GBEs was constructed *in situ* (Figure 1A)*.* A deletion of 30 to 123 amino acids (AA) was achieved by homologous recombination of pre-designed linear PCR products binding to the *glgB* gene [27]. In addition, a full *glgB* knockout strain (*E. coli* DH5α ΔglgB) was also constructed. Sequencing and alignment of *glgB* in each *E. coli* DH5α strain confirmed that no unanticipated mutations existed. However, insertion-deletion recombination leaves a scar sequence upstream of *glgB* with a new ribosome-binding site (RBS) (Figure 1B), which replaces the original regulatory region.

### Influences of N-terminal truncation of glgB on transcription of the glgBXCAP operon

Previously, glycogen metabolism genes in *E. coli* were considered to form two operons: *glgBX* and *glgCAP* [4]. It is now known that *glgBXCAP* consists of a single transcription unit with a sub-operon promoter within *glgC* directing the expression of *glgAP* [42]. Thus, manipulation of *glgB* may alter the expression, not only of *glgB*, but also the downstream genes, which can in turn affect glycogen structure and accumulation. In addition, the inserted scar sequence may also have unpredictable effects on *glgB* expression. Quantitative RT-PCR showed that the *glgB*, *glgX*, *glgC*, *glgA*, and *glgP* in all the six *E. coli* DH5α strains were expressed at 20 h except for the full *glgB* knockout strain. Expression levels, averaged over the two biological and two technical replicates, normalized to the level of *cysG*, can be found in Table 1. There was a good correlation between transcription levels over the five genes of the *glgBXCAP* operon from the WT versus the corresponding genes in each of the mutants: *E. coli* DH5α glgBΔ90 versus WT (r^2^ = 0.91), *E. coli* DH5α glgBΔ180 versus WT (r^2^ = 0.79), *E. coli* DH5α glgBΔ270 versus WT (r^2^ = 0.58), *E. coli* DH5α glgBΔ369 versus WT (r^2^ = 0.93) and *E. coli* DH5α ΔglgB versus WT (r^2^ = 0.79). The effect of the sub-operon promoter can also been clearly seen in the substantially increased expression of *glgC*, and to lesser extent, *glgA* and *glgP* (Table 1).

**Table 1:** **Level of Gene Express for** ***E. coli*** **DH5α strains, averaged over 2 technical and 2 biological replicates, normalized to the level of** ***cysG***

**Gene**	***WT***	***∆90***	***∆180***	***∆270***	***∆369***	***∆glgB***
*glgB*	1.20	3.71	9.32	11.20	3.86	0.08
*glgX*	0.68	1.66	3.83	4.07	2.42	7.83
*glgC*	6.07	8.34	13.51	12.19	10.96	16.55
*glgA*	2.08	1.71	4.19	5.18	2.82	4.86
*glgP*	2.82	3.52	6.99	5.68	3.44	3.85

### Influence of GBE N-terminus on glycogen accumulation

**Figure 2:**
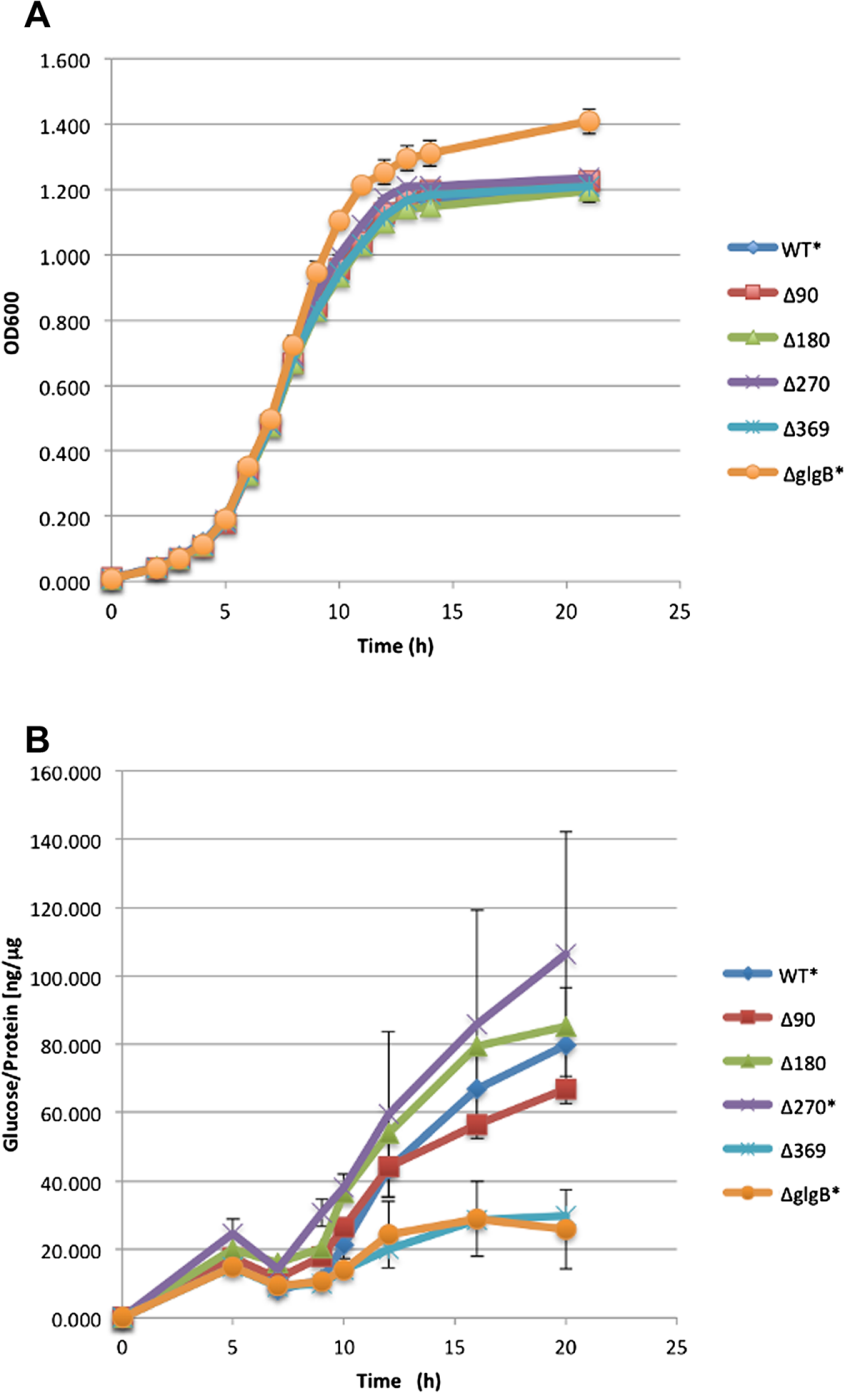
Growth of *E. coli* DH5α strains in 1 × M9 minimal media (T/G = 1:2) and corresponding glycogen accumulation. **A**. Cell density is plotted against time averaged over four independent OD_600_ readings. **B**. Glycogen accumulation is expressed as ratio of glucose to protein amount over time. Three independent replicates were performed. All data were presented as means ± standard error.

The amount of glycogen accumulation in the six *E. coli* DH5α was observed through corresponding growth curves (Figure 2). *glgB* manipulation did not change bacterial growth rates (Figure 2A). Since 1 × M9 (T/G = 1:2) is a minimal medium, bacterial density could only reach a cell density around 0.9-1.0 (OD_600_). A small peak of glycogen accumulation appeared at the 5th hour for all strains, and corresponded to the early exponential phase. After a further 3 hrs of incubation, glycogen levels dropped to their lowest levels in late exponential phase, presumably because the glucose was more needed for growth than glycogen accumulation. After reaching stationary phase at 9 hrs, glycogen accumulation was maintained for another 10 hrs.

### Alteration of glycogen structure due to GBE N-terminal truncation

The structure of the glycogen was further assessed using iodine staining and fluorophore-assisted carbohydrate electrophoresis (FACE). Iodine staining is a frequently used method for detecting glycogen content and structure in microorganisms grown on agar plates [17]. The intensity of the stain corresponds with ACL, whereby glycogen with short ACL stains a light-yellow in contrast to glycogen with long ACL which stains dark brown. *E. coli* DH5α and glgBΔ270 appears as light-yellow colonies while *E. coli* DH5α glgBΔ90, glgBΔ180, and glgBΔ369 were dark brown. In contrast, *E. coli* DH5α ΔglgB colonies were stained to dark blue, indicating the presence of amylose-like long linear α1,4-glucan polysaccharide (Additional file 1: Figure S1). λ-max scanning of glycogen-iodine complexes from each isolate over a wavelength range of 350-750 nm confirmed these conclusions (Additional file 1: Table S3) and concurred with previous findings [43]. The highest λ-max value of 585 nm was detected for strain ΔglgB, which is close to the absorbance of long linear α1,4-glucan amylose (~600 nm), as compared to 405 nm for the wild type strain, which is close to the absorbance maximum for glycogen (~420 nm). Thus according to the iodine vapor staining and λ-max scanning results, the N-terminal truncation of GlgB altered the glycogen structure of *E. coli* DH5α.

**Figure 3:**
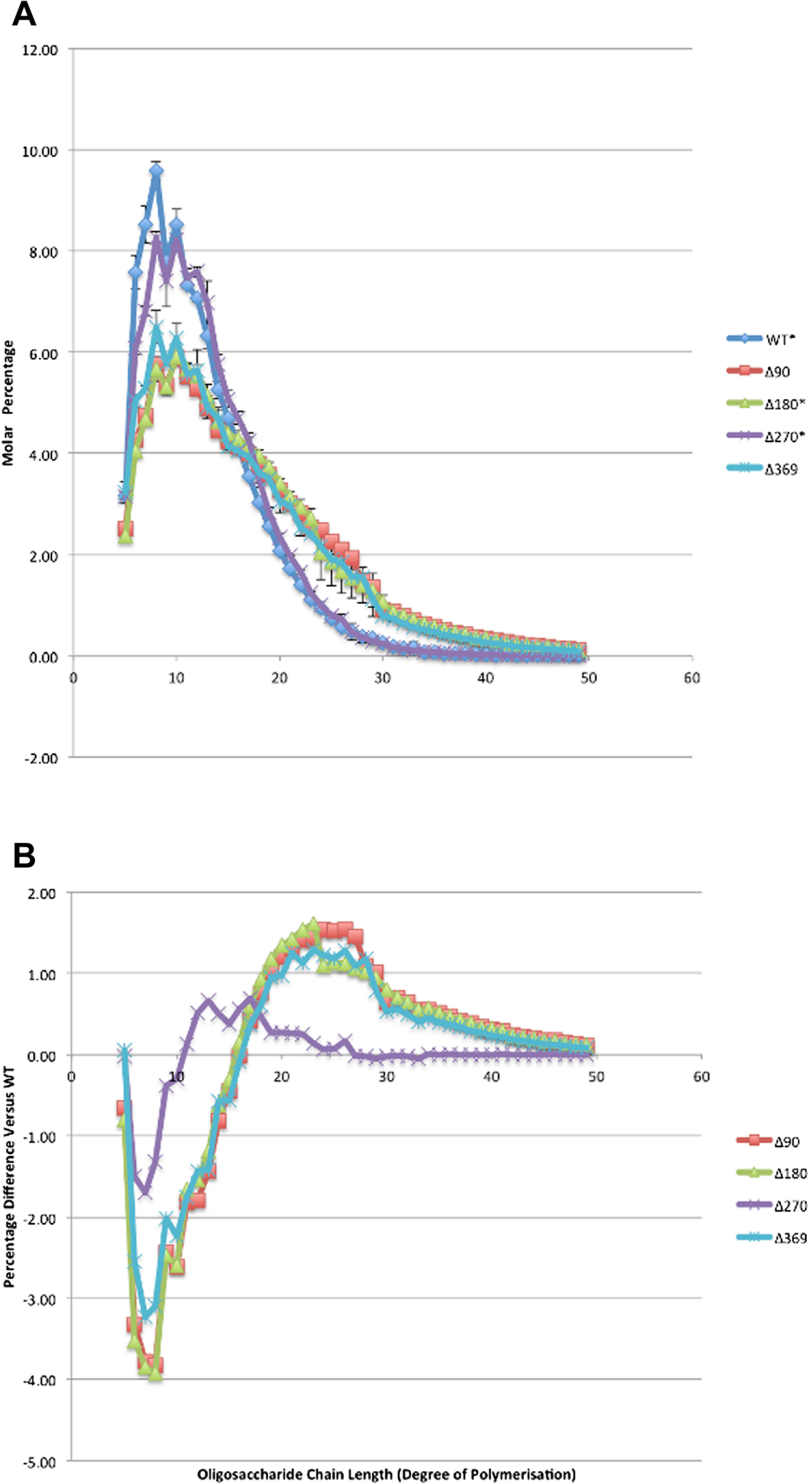
Chain length distributions of oligosaccharides in glycogen extracted from the six *E. coli* DH5α strains. **A**. Chain length distributions of isoamylase-debranched glycogen, which are expressed as molar percentage (%) in terms of oligosaccharide chain length. **B**. Difference plot generated by subtracting the molar percentage of the respective WT oligosaccharide DP from the corresponding glgBΔ90, glgBΔ180, glgBΔ270, and glgBΔ369 molar percentages. The experiment was performed twice independently with two repeats for each replicate.

FACE was used to compare chain length distributions of isoamylase debranched glycogen from the six *E. coli* DH5α strains (Figure 3A). No oligosaccharides greater than 49DP were detected in any of the strains except for *E. coli* DH5α ΔglgB, where no measurements were recorded at all because, consistent with this being an essentially linear, amylose-like α1,4-glucan molecule, the DP had gone out to a chain length which cannot be directly detected by the instrument. The capillary electrophoresis instrument used in this study can typically measure debranched α1,4-glucan from DP6-80 [44]. Hence, the novel long linear chain glycogen synthesized by ΔglgB has elongated to the point that it is already comparable to long linear chain amylopectin and amylose polymers [44], which we refer to here as ‘amylose-like’. This is consistent with the classification based on iodine vapor staining and the reducing end assay (discussed below). A molar difference plot of FACE (Figure 3B), in which the values obtained for the wild type strain are subtracted from those of the mutant strains, was used to determine whether there are any changes to the glycogen structures from the N-terminal deleted GlgB-expressing strains. In order to better understand the FACE results, glycogen ACL (see Table 2) was calculated for each strain, averaged over two independent experiments using the formula provided in the Materials and Methods section. The ACL for WT glycogen was 12.35 DP, standard deviation 0.30 DP. On that basis, 5-11 DP can be characterised as having shorter ACL, while chains 13 DP or greater can be characterised as having longer ACL. A significant reduction in the proportion of shorter chains and an increase in the proportion of longer chains were observed in most of the mutant strains compared to the wild type. The glycogen in glgBΔ90 had 34.1% short chains (versus 52.5% in the wild type strain) and 60.7% long chains (versus 40.4% in the wild type strain). For glycogen in glgBΔ180, the corresponding percentages were 33.7% short chains and 60.8% long chains, while the glycogen in glgBΔ369 had 37.7% short chains and 56.7% long chains. In a complete contrast to these, glycogen from glgBΔ270 had 47.4% short chains and 45.0% long chains which is not that different to the distribution in the wild type glycogen. The similarity between the glycogen from the glgBΔ270 strain and that from the parent, wild type strain is also evident in the molar percentage and molar difference plots.

**Table 2:** **Average chain length of glycogen extracted from each of** ***E. coli*** **DH5α strains based on FACE data, across 2 biological and, for each of these, 2 technical replicates**

***E. coli*** **DH5a strain**	**Average chain length (Glucosyl Residues)**		
	**Replicate 1**	**Replicate 2**	**Average**
**WT**	12.35	11.90	12.13
**glgBΔ90**	16.64	16.01	16.33
**glgBΔ180**	16.53	18.25	17.39
**glgBΔ270**	12.83	12.82	12.83
**glgBΔ369**	15.76	14.70	15.23

Reducing end assay of amylose-like ‘glycogen’ from *E. coli* DH5α ΔglgB demonstrated that the branching frequency in the polysaccharide was significantly reduced and as low as 6.7 nmol of maltotriose equivalent, while the branching frequency in the commercial oyster glycogen is 537.7 nmol of maltotriose equivalent (Additional file 1: Figure S2). This further confirms that the amylose-like glycogen of ΔglgB does not just have significantly longer ACL but it is also significantly less branched hence really comparable in structure to long chain amylopectin or amylose.

### Starvation survival assay

**Figure 4:**
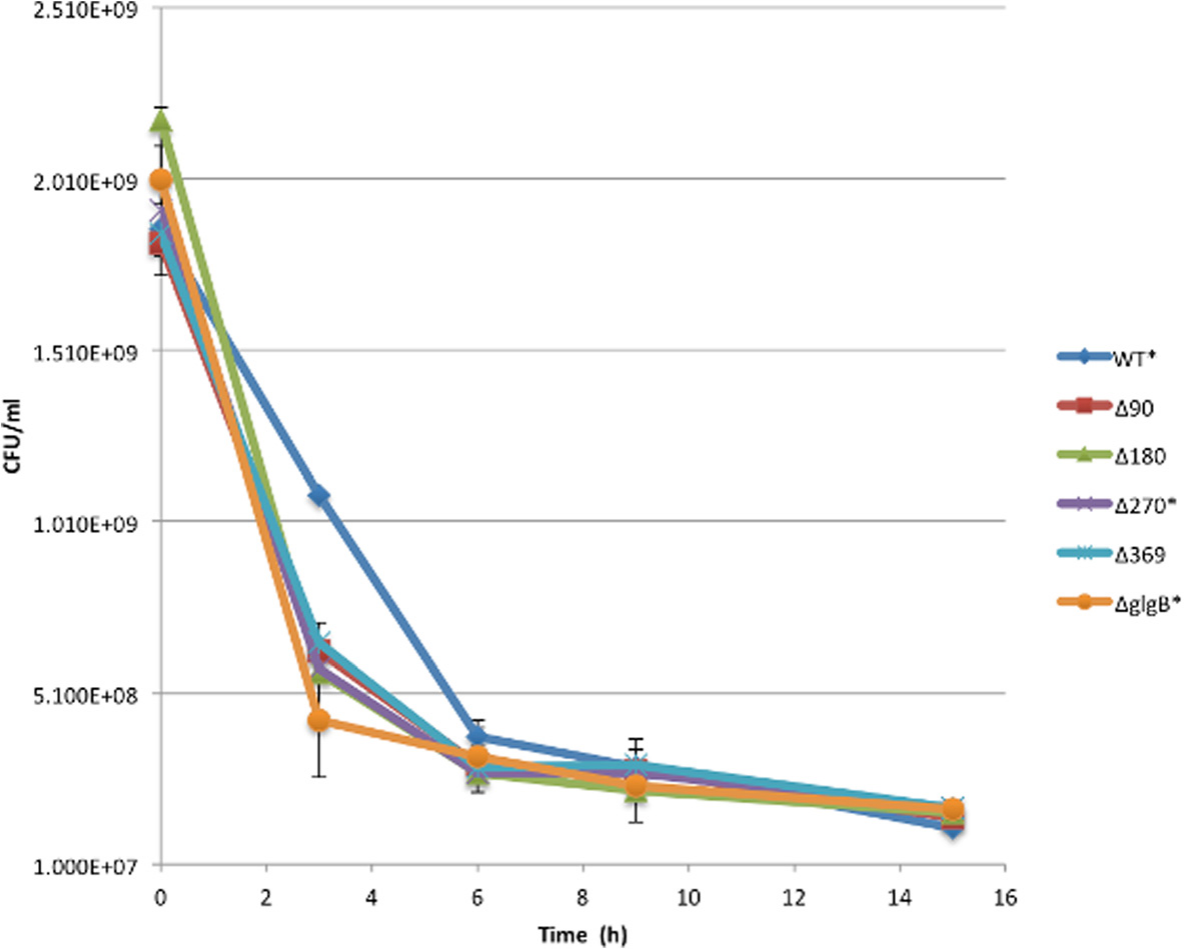
Starvation survival assay for E. coli DH5α strains in PBS buffer for 15 days. For each strain, two independent replicates were performed. Each replicate includes. four repeats. Viable cells of the six strains drop sharply for the first six days. *E. coli* DH5α survived better, especially at day 3, than *E. coli* DH5α ΔglgB from day 0 to 9. The other four strains behaved similar with no obvious difference. After day 6, cells died at a very slow rate. At day 15, the number of colony-forming units (CFU) for the six E. coli DH5α strains converged together.

To study the influence of GBE N-terminal truncation on *E. coli* starvation survival, strains were suspended in PBS buffer for 15 days. Colony forming unit counts were recorded to create a starvation survival curve (Figure 4). At day 3, *E. coli* DH5α wild-type was down to 58.4% of the starting count, but the mutated stains fared considerably worse, with viabilities down to 34.7% (glgBΔ90), 26.0% (glgBΔ180), 30.3% (glgBΔ270), 35.5% (glgBΔ369) and 21.5% (ΔglgB). However, at the day 6 mark all the strains had similar viability to the parent strain (12.6%-16.5% for the mutated strains versus 20.5% for the wild type).

### Desiccation resistance

**Figure 5:**
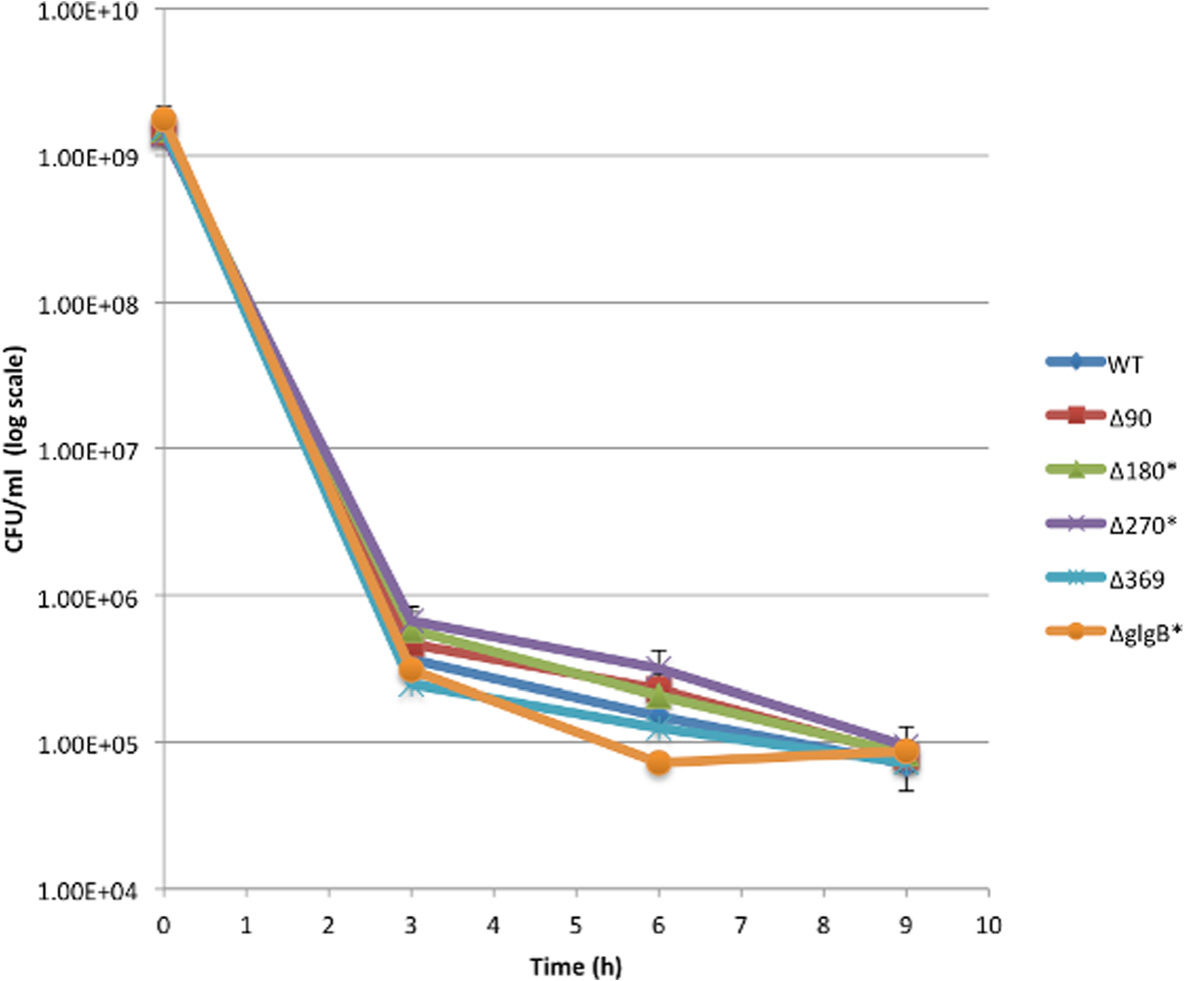
Desiccation survival abilities of E. coli DH5α strains. Counts of colony-forming units (CFU) are plotted for each strain at time point 0 h, 3 h, and 6 h. Two independent biological replicates were performed for each strain at each time point. For each replicate, four technical repeats were included.

Since glycogen can be converted to trehalose [12], we considered whether the differences in glycogen structure within the mutants would affect the trehalose production pathway. To test this hypothesis, we investigated desiccation tolerance, which has been shown to be dependent upon trehalose accumulation [13,45]. Both the wild type and mutant strains died rapidly in the first 3 hrs. The wild type strain CFU count was 0.024% of the starting value. The mutated strains had similar counts. The counts halved again in the next 6 hours. More importantly, there was little difference between wild type and most of the mutated strains, though ΔglgB fared worse than the other strains, most clearly at the 6 hr time point (Figure 5)

.

### Cold stress assay

The situation with the desiccation stress assay was mirrored in the cold stress assay, with all the strains dying rapidly, though once again ΔglgB fared worse than the other strains at the 6 hr time point despite starting with a greater CFU count at the outset. The glgBΔ369 strain also showed this pattern (Additional file 1: Figure S5).

### Biofilm formation assay

**Figure 6:**
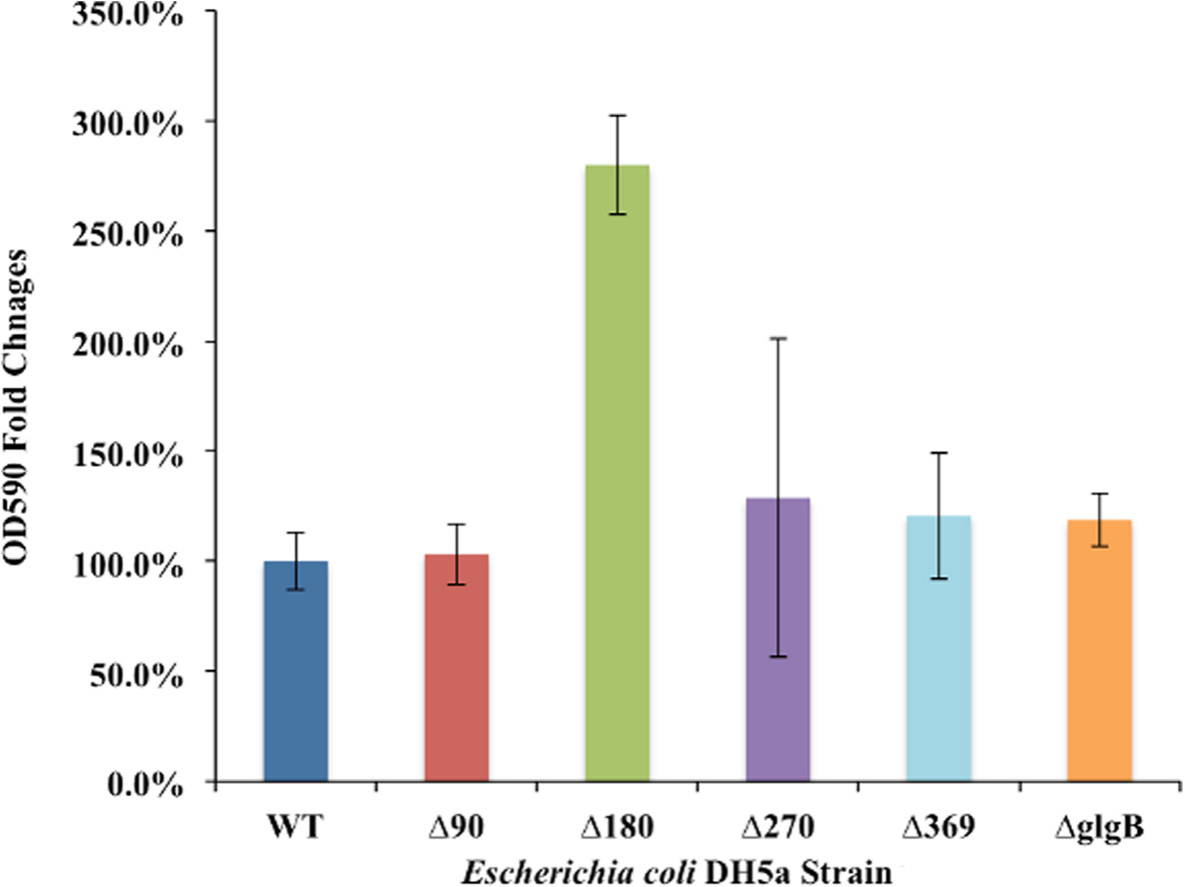
Quantification of biofilm formation abilities of *E. coli* DH5α strains on 96-well polystyrene plates in LB. Crystal violet staining method is used. Findings are expressed as fold changes versus WT based on based on average of three independent replicates. Bacteria in LB broth had enhanced biofilm formation abilities compared to bacteria growing in M9 (T/G = 1:2), where biofilm formation abilities were uniform (including Δ180), but a quarter of the level of that seen for the strains cultured in LB broth (Data not shown).

Two types of media, LB and 1 × M9 minimal medium (T/G = 1:2), were used for comparison. For *E. coli* DH5α strains cultured in 1 × M9 minimal medium no significant difference was observed among the strains (data not shown). On the other hand, comparison of LB and 1 × M9 minimal medium (T/G = 1:2) showed that LB broth greatly improves bacterial biofilm formation abilities, though this is to be expected given the nature of the two media. Overall, the biofilm forming abilities of wild type *E. coli* DH5α and the ΔglgB strain were not significantly different (given experimental errors), across 3 repeats involving 2 different media. However, point for point across the three experiments the biofilm forming capabilities of the ΔglgB strain were greater than that of the wild type strain, which is significant based on a two tail binomial distribution statistic. Finally, the most surprising result was that *E. coli* DH5α glgBΔ180 in LB broth showed a significant increase in biofilm formation (Figure 6).

## Discussion

The N-terminus of *E. coli* GBE influences the chain length distribution of glycogen, consistent with the previous *in vitro* study [23]. However, unlike the earlier *in vitro* experiment [23], no strong correlation between progressive shortening of N-terminus and gradual increase of transferred oligosaccharide chains was observed. Our results (Figure 3A) showed that glycogen samples from *E. coli* DH5α glgBΔ90, *E. coli* DH5α glgBΔ180, and *E. coli* DH5α glgBΔ369 have similar glycogen chain length distribution patterns (also reflected in similar ACLs in Table 2), while glycogen from *E. coli* DH5α glgBΔ270 has a distribution pattern that is between the wild type and the other mutant strains, with a reduced percentage of 5-10DP oligosaccharides and increased percentage of 11-26 DP oligosaccharides (Figure 3B). No difference in chain length distribution was observed beyond 26DP between the *E. coli* DH5α glgBΔ270 and the wild type strain.

The observed difference of chain length distributions caused solely by N-terminal truncated GBEs in *in vitro* study [23] is probably compensated by enzymes such as GlgP and GlgX *in vivo*. Chain length distribution patterns of glycogen samples indicate that the six *E. coli* DH5α strains can be divided into three groups: *E. coli* DH5α (ACL = 12.13DP) and *E. coli* DH5α glgBΔ270 (ACL = 12.83DP) form Group 1, *E. coli* DH5α glgBΔ90 (ACL = 16.33DP), *E. coli* DH5α glgBΔ180 (ACL = 17.39DP), and *E. coli* DH5α glgBΔ369 (ACL = 15.23DP) belong to Group 2, and finally *E. coli* DH5α ΔglgB (ACL not available due to its linear structure) forms Group 3.

In this study, we confirm that, apart from glycogen content, glycogen structure also has an impact on iodine staining (Additional file 1: Figure S1). An earlier study only focused on the relationship between amylose chain length and iodine staining [46], according to which longer chain length leads to absorbance at comparatively higher wavelengths (λ-Max). Iodine vapor staining has frequently been used to detect glycogen content, while glycogen structure (ACL) was largely ignored [16];Lerner, 2009 #1353}, although Dauvillee et al. [17] mentions that iodine staining can provide information about the structure of glycogen or starch. In this study, we showed that λ-Max of glycogen-iodine solution (Additional file 1: Table S3) is related to glycogen ACL (Table 2) with longer ACL generating darker brown colour. Moreover, the classification of *E. coli* DH5α strains based on iodine vapor staining (Additional file 1: Figure S1) is consistent with the classification by FACE (Figure 3).

Unexpectedly, the strain *E. coli* DH5α glgBΔ270 exhibited a significant level of GBE activity compared to other mutants. Despite having 39 residues truncated at N-terminus there were minimal changes in chain transfer pattern and substrate preference [47]. It had been assumed that the 39 residues are without specific function. It was also suggested that the activity of GlgB is reduced in proportion to the number of amino acids truncated, based on the study of three truncated GlgBs (GlgBΔ112, Δ121, and Δ171) from *M. tuberculosis H37Rv* [48]. As mentioned earlier, such a relationship was not observed in the current study, which requires further exploration for an accurate explanation. A potential explanation is that some N-terminal truncations lead to previously unidentified and beneficial foldings for GBE so that it became catalytically more active compared to the other N-terminal truncated GBEs. A recent study also found that four enzymes in deoxyxylulose phosphate pathway (DXP) are highly insoluble, which affects metabolite formation, while increased solubility of enzymes provides a ‘strategy to increase the production of secondary metabolites’ [49]. Thus, N-terminal truncation induced enzyme solubility may be one of reasons for the enzyme’s functional improvement.

Bacterial GBEs can be divided into two groups according to the lengths of their N-terminus [24]. We collected a set of 1035 GBE sequences belonging to different bacterial species from Uniprot database [50] to determine the distribution of GBE lengths, and found that two apparent peaks exist with about 100 AA difference (see Additional file 1: Figure S3). These peaks are in agreement with earlier findings that GBEs can be divided into Group 1 (both N_1_ and N_2_ modules present) and Group 2 (only N_2_ module present), according to the composition of N-terminus [51]. In addition, the N_1_ module is hypothesized to originate from the duplication of N_2_ module [51]. Recently the N_2_ module has been re-annotated as a carbohydrate-binding module (CMB48) [20], while the function of the N_1_ module is still not clear. Interestingly, the N_1_ module in *E. coli* is defined as the first 106 AA residues [24,51]. However, an alignment of the Pfam hidden Markov model (HMM) CMB_48 [52] starts at position 124, suggesting that N_1_ could span as much as the first 123 AA.

In any case, although a structure has been determined for *E. coli* GlgB (1m7x) it only starts at residue 113; the full-length protein could not be crystalised and the structure of the N-terminal residues remains undefined [53].

Because the amount of glycogen accumulation is an important factor for bacterial survival [6], the influence of N-terminal truncation of GlgB on glycogen accumulation was also investigated by measuring glycogen content in cells (Figure 2). Growth curves show no significant differences among strains (Figure 2A). Thus, *glgB* is not essential for bacterial growth [54] because its complete abolition is not lethal. However, the N-terminus of GlgB is involved in glycogen accumulation (Figure 2B). Ranking of glycogen content from high to low amount at 20 h for the six strains is *E. coli* DH5α glgBΔ270, followed by *E. coli* DH5α glgBΔ180, *E. coli* DH5α wild type,*E. coli* DH5α glgBΔ90, *E. coli* DH5α glgBΔ369, and *E. coli* DH5α ΔglgB. A previous report has confirmed that *glgB*-deficient *E. coli* accumulates a very low-level of glycogen [55]. By also looking at Table 1 (top row), it is interesting to see that glycogen amount is highest in *E. coli* DH5α glgBΔ270, which corresponds to the strain with the highest transcription of the corresponding N-truncated gene. *E. coli* DH5α glgBΔ180 follows this pattern, but then *E. coli* DH5α glgBΔ90 and wild type *E. coli* DH5α do not quite follow the pattern and *E. coli* DH5α glgBΔ369 is out of place with a higher level of expression that *E. coli* DH5α glgBΔ90 or wild type *E. coli* DH5α.

The glycogen accumulation ability of *E. coli* DH5α glgBΔ369 is observed to be comparatively low and very close to that of *E. coli* DH5α ΔglgB, indicating that GBE in *E. coli* DH5α glgBΔ369 is less functional compared to other N-terminal truncated strains. Specifically, GBEs in the other three N-terminal truncated strains are only manipulated in N1 module while N2 module could still replace N1 module for essential functions, considering that N1 module is originated from duplication of N2 module. However, for GBE in *E. coli* DH5α glgBΔ369, both N1 and N2 modules are potentially affected. Thus, no compensation mechanism is available for this truncated GBE. However, since we can still detect chain length distribution for glycogen in this strain, GBE in *E. coli* DH5α glgBΔ369 still retains some of its branching activity.

Our original purpose was to alter glycogen ACL solely by manipulating GBE N-terminus in order to test the influences of glycogen ACL on bacterial durability in face of abiotic stress. However, by truncating GBE N-terminus, we found that glycogen ACL and accumulation are inter-related, which leads to the difficulty in drawing a definite conclusion about which is the major factors influencing bacterial durability. In our results, we did not see any difference among the four N-terminal truncated strains in terms of starvation resistance (Figure 4). However, compared to the survival rate of the wild type strain, GBE N-terminal truncation damaged bacterial starvation ability. That said, the deletion strain fared worst, suggesting that possessing glycogen, rather than amylose-like polysaccharide, is an advantage. It is intriguing that the highest level of glycogen with comparatively short ACL in *E. coli* DH5α glgBΔ270 does not show any advantage in starvation survival. For desiccation resistance, *E. coli* DH5α glgBΔ90, *E. coli* DH5α glgBΔ180, and *E. coli* DH5α glgBΔ270 show higher ability than *E. coli* DH5α (see Figure 5), which may be ascribed to faster degradation of glycogen due to longer glycogen ACLs, though this explanation does not account for glgBΔ270. The released glucose may be used for the synthesis of trehalose for stress protection. Although glycogen ACL in *E. coli* DH5α glgBΔ369 is also longer than that in *E. coli* DH5α, the amount of glycogen is much lower in this strain. In addition, glycogen content itself has also been linked with stress resistance ability regardless of the impairment of glycogen degradation pathway [5,25]. High glycogen content results in a significant increase in intracellular water volume, which is beneficial for bacterial desiccation survival [56]. While our studies have shown that N-terminal truncation enhances bacterial desiccation resistance, depending on both glycogen ACL and amount, further investigation is required to tease out the individual effects of glycogen structure and content. Here again, the deletion strain fared worst, a pattern we also noted in the low temperature (4°C) viability assays.

Finally, biofilm formation was assayed across the set of strains. Biofilm formation is widely recognized as an important strategy for bacteria to survive and persist in harsh environmental conditions [26]. Cells are normally enclosed in a polymer matrix consisting of DNA, protein, and polysaccharides [57]. A study of *Salmonella enteritidis* demonstrated that “biofilm was formed from glycogen cell stores” [8]. A recent study also showed that *glgP* deficiency compromises biofilm formation of *Azospirillum brasilense* Sp7 [25]. The global regulatory protein *csrA*, that is involved in glycogen biosynthesis, is also confirmed to control the formation and dispersal of biofilms in *E. coli* [58]. Since N-terminal truncation of GlgB changes glycogen ACL and accumulation, we decided to investigate whether GlgB N-terminal truncation has any influence on *E. coli* DH5α biofilm formation ability. Bacteria in LB broth had enhanced biofilm formation abilities compared to bacteria growing in M9 (T/G = 1:2), where biofilm formation abilities were uniform, but at a quarter of the level of that seen for the strains cultured in LB broth. This is supported by a previous study that showed that glucose inhibition of biofilm formation is common for *Enterobacteriaceae* genus [59]. In contrast to our observations based on *E. coli*, a positive correlation between glycogen concentration and biofilm formation has been reported in *Salmonella enteritidis* SE3934 [8]. Thus, the effects of glucose on biofilm formation seem to vary in different microbial species, though the medium in which the bacteria are grown is clearly a factor. Biofilm forming ability was also uniform across the LB broth experiments, except for *E. coli* DH5α glgBΔ180, which showed significantly enhanced biofilm formation ability. This is noteworthy, but requires further investigation to determine the underlying mechanisms. However, with the exception of the glgBΔ180 result in LB broth, the fact that the biofilm forming ability of ΔglgB strain was at the same level as the other strains suggests that it is the presence of a long chain polysaccharide that is important for biofilm formation, rather than its structure. One final comment is appropriate at this point: we have, arguably, extracted as much as one can from *E. coli* DH5α, and now propose that more unequivocal results would have been obtained with a less labile starting species.

## Conclusions

Despite *glgB* being the first gene of an operon, it is clear that *in situ* mutation is a viable means to create more biologically relevant mutant strains. Secondly, there was the suggestion in the data that impairments of starvation, cold and desiccation resistance were worse for the strain lacking *glgB*, though the first of these was not statistically significant. The results provide prima facie evidence linking abiotic stress tolerance with shorter glycogen ACL. However, further work needs to be done, perhaps in a less labile species. Further work is required to establish why glgBΔ270 was efficient at producing shorter ACL glycogen, and why the strain glgBΔ180 showed increased ability to form biofilms. Finally, further work is also required to tease out the complex relationship between glycogen abundance and glycogen structure.

## Additional file

Additional file 1: Figure S1. Results of iodine vapor staining of *E. coli* DH5α colonies growing on M9 minimal agar plates. a-f represent the six *E. coli* DH5α strains as annotated above. Based on the staining color, we divided the six strains into three groups arbitrarily. a and d belong to group 1 with light yellow-brownish color. b, c, and e are classified as group 2 with dark brownish color. f belongs to group 3 with dark blue color. **Figure S2.** Comparison of reducing ends in four polysaccharides: amylose, amylopectin, oyster glycogen, and glycogen from full *glgB* knockout strain *E. coli* DH5α ΔglgB. Three replicates were performed for each sample. **Figure S3.** Length distribution of bacterial GBEs. 1035 GBEs were selected from UniProt database under the name of “glycogen branching enzyme” or “1,4-alphaglucan branching enzyme”. Each item was from a unique bacterial species while redundant or fragment sequences were removed manually or by nrdb90.pl. R was used to plot the histogram. Two major GBE groups can be identified in the figure, which shows consistent pattern with the result of Lo Leggio et al [53]. **Figure S4.** Cold stress (4oC) tolerance assay. Strains were sampled at days 3, 6, 10 and 13, with 3 replicates at each data point. Error bars are +/- 1 standard deviation. **Table S1.** Bacterial strains used in this study. **Table S2A.** List of primers used in this study. **Table S2B.** List of primer pairs for the respective glgB deletion mutant stains. **Table S3.** Results of λ-max scanning (350 nm-750 nm) of glycogen-iodine solutions.

## Abbreviations

ACL: Average chain length
FACE: Fluorophore-assisted carbohydrate electrophoresis
GBE: Glycogen branching enzyme
