# Supplementary material for: Influence of in situ progressive N-terminal is still controversial truncation of glycogen branching enzyme in Escherichia coli DH5α on glycogen structure, accumulation, and bacterial viability
Source: BMC Microbiol. 2015 May 7;15:96. doi: 10.1186/s12866-015-0421-9 (PMC4433092; doi:10.1186/s12866-015-0421-9)
Supplement: Additional file 1: Figure S1. — Results of iodine vapor staining of E. coli DH5α colonies growing on M9 minimal agar plates. a-f represent the six E. coli DH5α strains as annotated above. Based on the staining color, we divided the six strains into three groups arbitrarily. a and d belong to group 1 with light yellow-brownish color. b, c, and e are classified as group 2 with dark brownish color. f belongs to group 3 with dark blue color. Figure S2. Comparison of reducing ends in four polysaccharides: amylose, amylopectin, oyster glycogen, and glycogen from full glgB knockout strain E. coli DH5α ΔglgB. Three replicates were performed for each sample. Figure S3. Length distribution of bacterial GBEs. 1035 GBEs were selected from UniProt database under the name of “glycogen branching enzyme” or “1,4-alphaglucan branching enzyme”. Each item was from a unique bacterial species while redundant or fragment sequences were removed manually or by nrdb90.pl. R was used to plot the histogram. Two major GBE groups can be identified in the figure, which shows consistent pattern with the result of Lo Leggio et al [53]. Figure S4. Cold stress (4oC) tolerance assay. Strains were sampled at days 3, 6, 10 and 13, with 3 replicates at each data point. Error bars are +/- 1 standard deviation. Table S1. Bacterial strains used in this study. Table S2A. List of primers used in this study. Table S2B. List of primer pairs for the respective glgB deletion mutant stains. Table S3. Results of λ-max scanning (350 nm-750 nm) of glycogen-iodine solutions. [file 12866_2015_421_MOESM1_ESM.pdf]

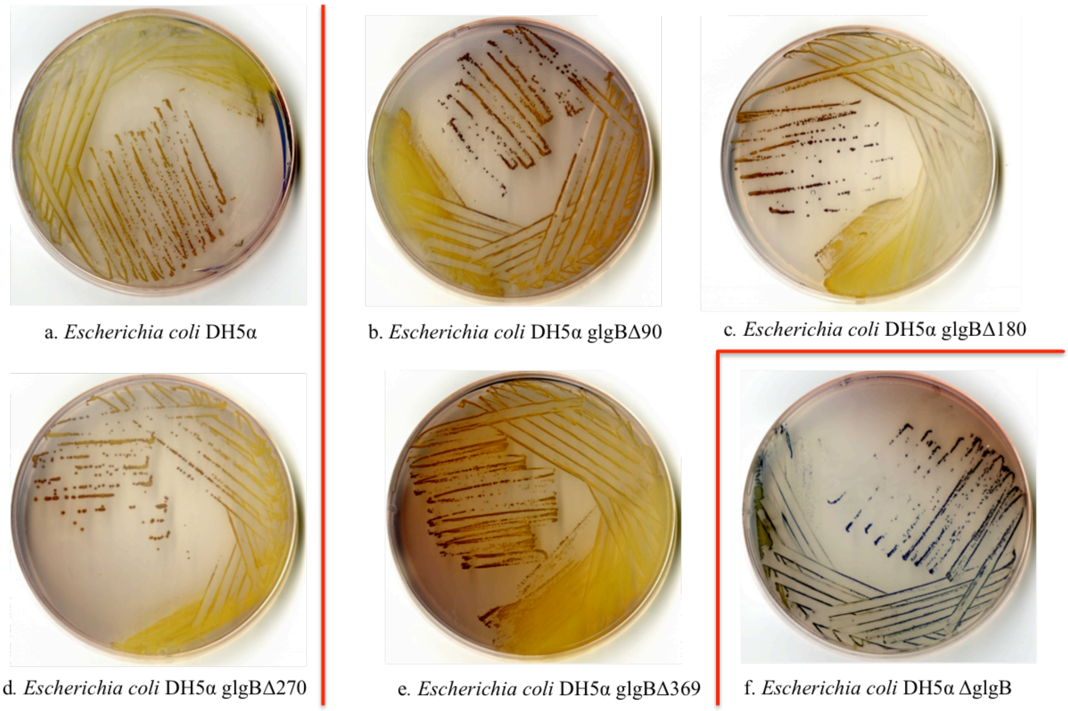

FIG S1. Results of iodine vapor staining of *E. coli* DH5α colonies growing on M9 minimal agar plates. a-f represent the six *E. coli* DH5α strains as annotated above. Based on the staining color, we divided the six strains into three groups arbitrarily. a and d belong to group 1 with light yellow-brownish color. b, c, and e are classified as group 2 with dark brownish color. f belongs to group 3 with dark blue color.

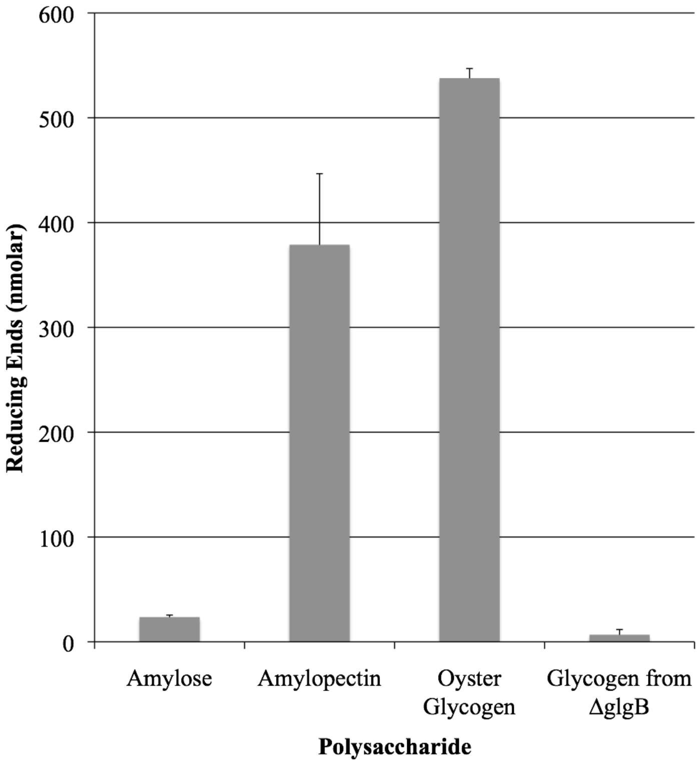

FIG S2. Comparison of reducing ends in four polysaccharides: amylose, amylopectin, oyster glycogen, and glycogen from full *glgB* knockout strain *E. coli* DH5α ΔglgB. Three replicates were performed for each sample.

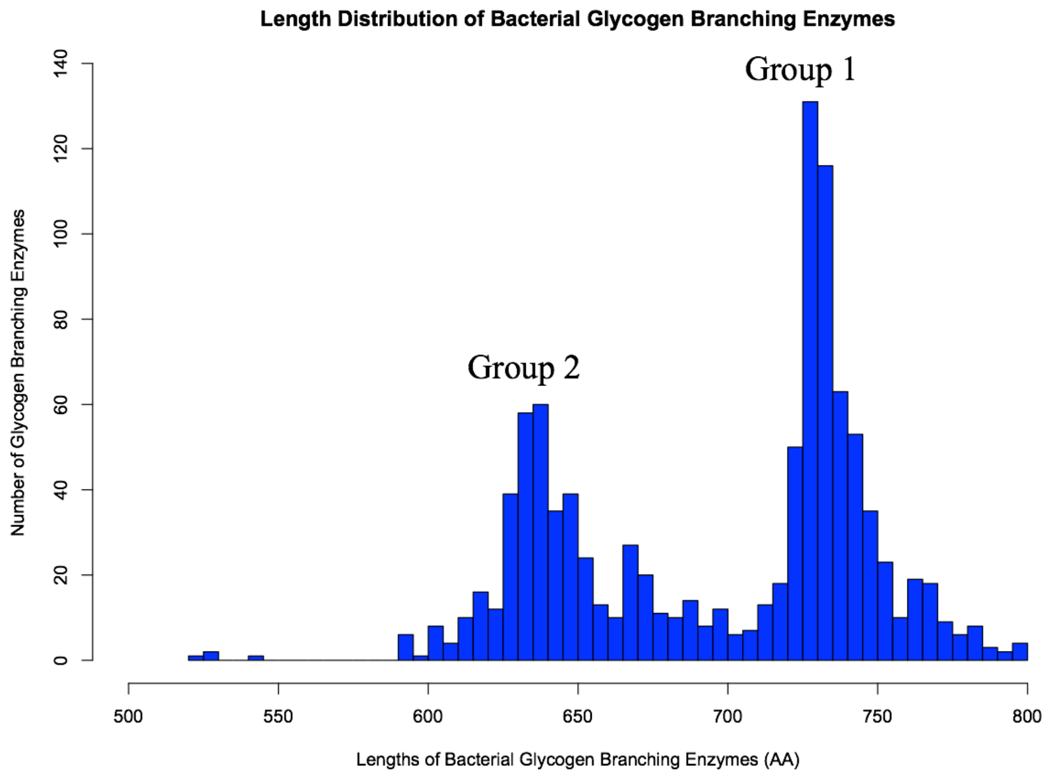

FIG S3. Length distribution of bacterial GBEs. 1035 GBEs were selected from UniProt database under the name of "glycogen branching enzyme" or "1,4-alpha-glucan branching enzyme". Each item was from a unique bacterial species while redundant or fragment sequences were removed manually or by nrdb90.pl. R was used to plot the histogram. Two major GBE groups can be identified in the figure, which shows consistent pattern with the result of Lo Leggio et al (53).

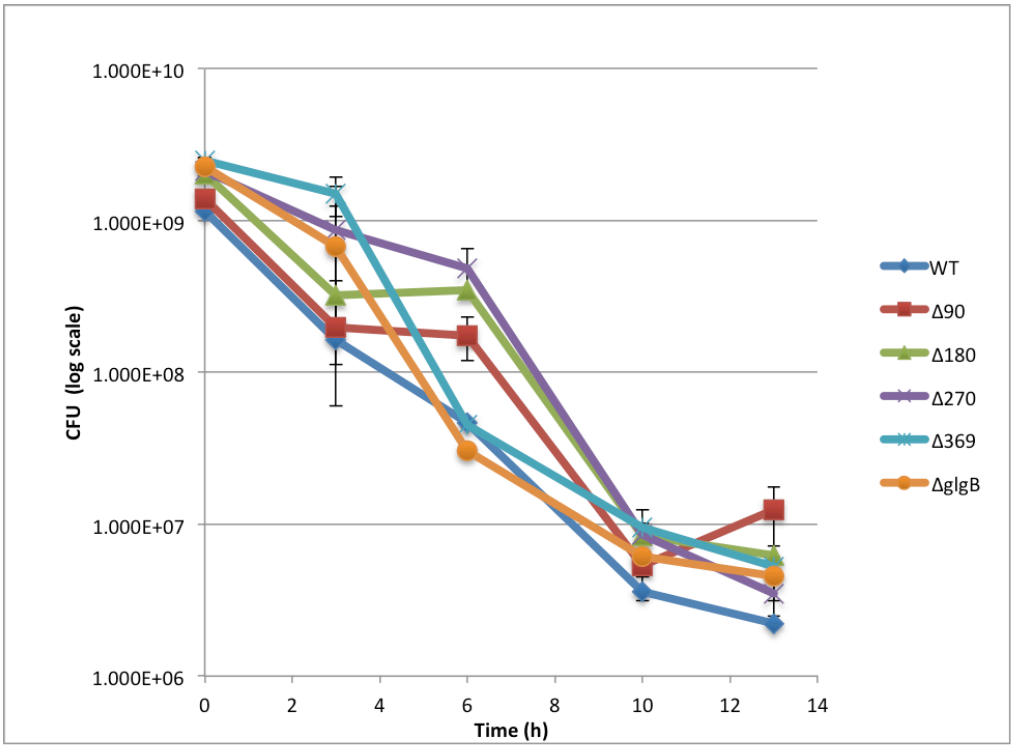

FIG S4. Cold stress (4°C) tolerance assay. Strains were sampled at days 3, 6, 10 and 13, with 3 replicates at each data point. Error bars are +/- 1 standard deviation.

TABLE S1. Bacterial strains used in this study

Bacterial Strain	Description	Sources
E. coli DH5α	Wild type	Invitrogen
E. coli DH5αΔglg90	90 bp deletion of glgB 5'	This study
E. coli DH5α glgΔ180	180 bp deletion of glgB 5'	This study
E. coli DH5α glgΔ270	270 bp deletion of glgB 5'	This study
E. coli DH5α glgΔ369	369 bp deletion of glgB 5'	This study
E. coli DH5α ΔglgB	Deletion of glgB	This study
E. coli BL21(DE3)	Wild type	New England Biolabs
E. coli JM109	Wild type	New England Biolabs
E. coli DB3.1	Wild type	Invitrogen
E. coli Top10	Wild type	Invitrogen

TABLE S2A. List of primers used in this study

Primer	5' – Primer Sequence – 3'
H1P1	TTGGATGGGGTGACACAATAAAACAGGAAGACAAGCGTGTAGGCTGGAGCTGCTTC
H2P2	GTCGGGTAAAAGGGCACGGACTTCCAGTCCCGCGGTCATATGAATATCCTCCTTAGT
H2P3	GACGCCGCTAAAGAATCCCCGTGAGTCGAGACACTCCATATGAATATCCTCCTTAGT
H2P4	ACCAAAACGGTAAGGATCATCAATCAGGTTTTGCTGCATATGAATATCCTCCTTAGT
H2P5	GACGCCATCCATAGTATCTGCATGCGCGCCTAAGGTCATATGAATATCCTCCTTAGT
H2P6	GCCGAGGGGAGCGGGTTTGCCAATGGCGAGTTGTGTCATATGAATATCCTCCTTAGTT
K_t	CGGCCACAGTCGATGAATCC
K_2	CGGTGCCCTGAATGAACTGC
FP*glgB*	CAATAAAACAGGAAGACAAGC
RP*glgB*	CCAATGGCGAGTTGTGTCAT
glgBF	TCACCTACCGCCACCATAAA
glgBR	GTTCTTTGTCATCCACCACCA
glgXF	CAACCATAAACACAATGAAGCAAAC
glgXR	CAAGAGAACCGCCTAACCCT
glgCF	ATCGTGCTTGTGTTATTCCG
glgCR	TATCGCTCCTGTTTATGCCC
glgAF	GAAGTCGGTGTTTACTGTGC
glgAR	GAAAGAGATTTGTCCGTTGAAT
glgPF	CTACGATAACCTGGCGGATAAA
glgPR	GACCTGACAACACACCTCAA
cysGF	TTGTCGGCGGTGGTGATGTC
cysGR	ATGCGGTGAACTGTGGAATAAACG

TABLE S2B. List of primer pairs for the respective glgB deletion mutant stains

Strain	Primer Pair	
E. coli DH5α glgBΔ90	H1P1	P2H2
E. coli DH5α glgBΔ180	H1P1	P2H3
E. coli DH5α glgBΔ270	H1P1	P2H4
E. coli DH5α glgBΔ369	H1P1	P2H5
E. coli DH5α ΔglgB	H1P1	P2H6

TABLE S3. Results of λ-max scanning (350 nm-750 nm) of glycogen-iodine solutions

**Glycogen Source**	**λ-Max (nm) Replicate 1**	**λ-Max (nm) Replicate 2**
E. coli DH5a	405	405
E. coli DH5a glgΔ90	530	530
E. coli DH5a glgΔ180	525	530
E. coli DH5a glgΔ270	410	395
E. coli DH5a glgΔ369	525	530
E. coli DH5α ΔglgB	585	585
